# Ultrasound Pattern of Intramuscular Polymethylmethacrylate in Gluteal Augmentation

**DOI:** 10.1111/jocd.70415

**Published:** 2025-08-25

**Authors:** Eliza Porciuncula Justo Ducati, Ximena Wortsman, Gabriel de Sousa Lima, Camila Souza de Araujo Guarinello

**Affiliations:** ^1^ ED Ultrassonografia Dermatológica Sao Paulo SP Brazil; ^2^ IDIEP ‐ Institute for Diagnostic Imaging and Research of the Skin and Soft Tissues Las Condes Santiago Chile; ^3^ Blanc Hospital Sao Paulo SP Brazil

**Keywords:** augmentation, cosmetic surgery, Polymethyl Methacrylate, ultrasound

To the Editor,

Polymethyl methacrylate (PMMA) microspheres have gained popularity as a permanent filler for gluteal augmentation due to their biostimulatory properties. Although ultrasonography is routinely employed to evaluate dermal fillers, descriptions of PMMA's intramuscular ultrasound signature remain scarce. Herein, we report the characteristic sonographic features observed in 40 female patients who underwent intramuscular PMMA injections, highlighting the diagnostic utility of high‐resolution ultrasound in this context.

We conducted an observational, descriptive study at a dermatological ultrasound clinic in São Paulo, Brazil, between June 2023 and October 2024. Approved by the Research Ethics Committee (CEP), number 0398, at Blanc Hospital São Paulo. Inclusion criteria comprised female patients aged 20–65 years who received PMMA (30% concentration; Biossimetric or Linnea Safe) in the gluteus maximus and/or medius 1 month prior. Exclusion criteria included autoimmune disorders, pregnancy, or injection of other substances in the gluteal region. Ultrasound examinations utilized a GE LOGIQ machine with both 6–15 MHz linear and 4–6 MHz convex transducers. The gluteal area was divided into four quadrants, and eight representative cases were selected to illustrate typical findings.

Ultrasonographically, subcutaneous PMMA deposition appeared as ill‐defined, hyperechoic regions with spiculated margins and numerous punctate echoes generating comet‐tail reverberations. Posterior acoustic shadowing was consistently observed due to high attenuation of the filler [1, 2]. In the muscular plane, PMMA induced diffuse echogenic enhancement and obliteration of the normal fibrillar pattern of muscle fibers. High‐frequency linear imaging (6–15 MHz) delineated loss of muscle fascicle definition beneath the hyperechoic fascia, whereas convex transducers (4–6 MHz) confirmed these changes at greater depth (Figures 1, 2 and 3).

**FIGURE 1:**
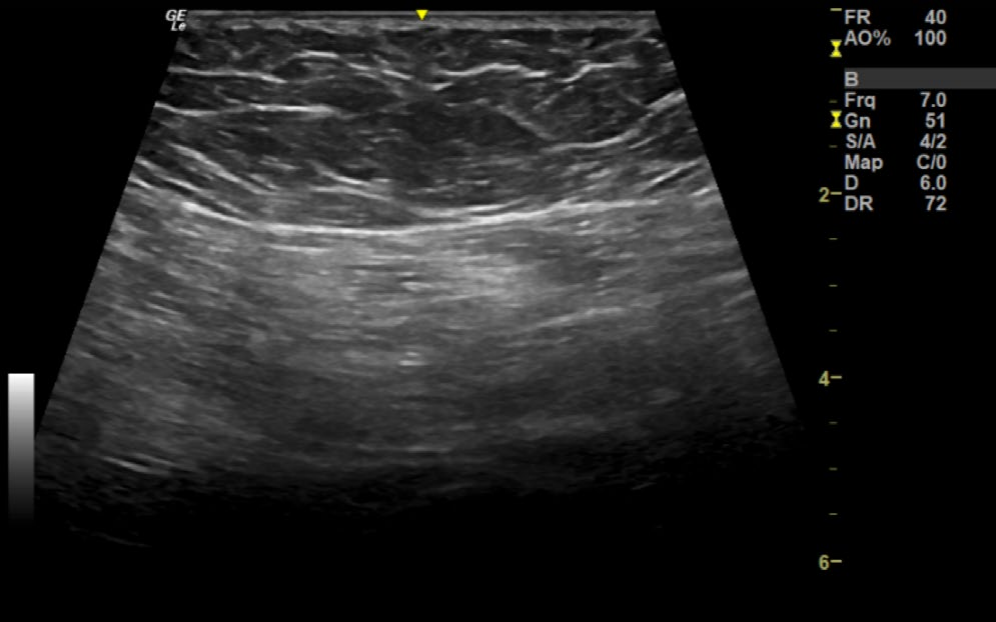
Intramuscular PMMA in the gluteus maximus observed in this image in the longitudinal axis with a linear transducer (6–15 mHz). Preserved subcutaneous tissue with a hypoechoic ultrasound pattern with hyperechoic lines (usual pattern of subcutaneous tissue without fillers). The echogenicity of the musculature (below the muscle fascia) is increased and there is a loss of the usual fibrillar pattern.

**FIGURE 2:**
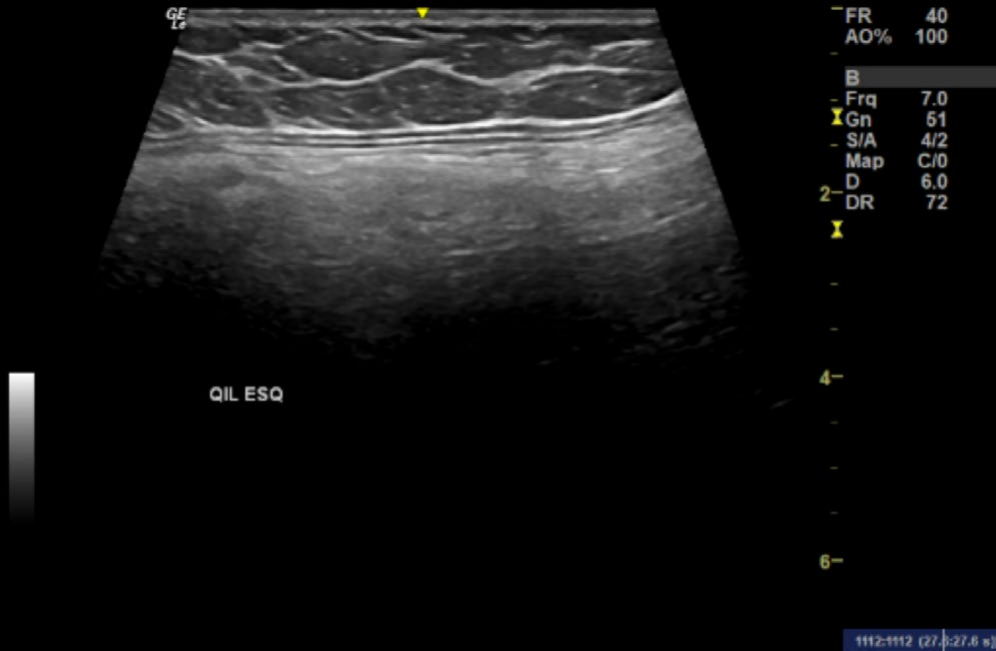
Image acquired in the longitudinal axis with a linear transducer (6–15 mHz) observing a global increase in the echogenicity of the gluteal muscles below the muscle fascia (hyperechogenic line) due to the presence of exogenous filler within the muscle.

**FIGURE 3:**
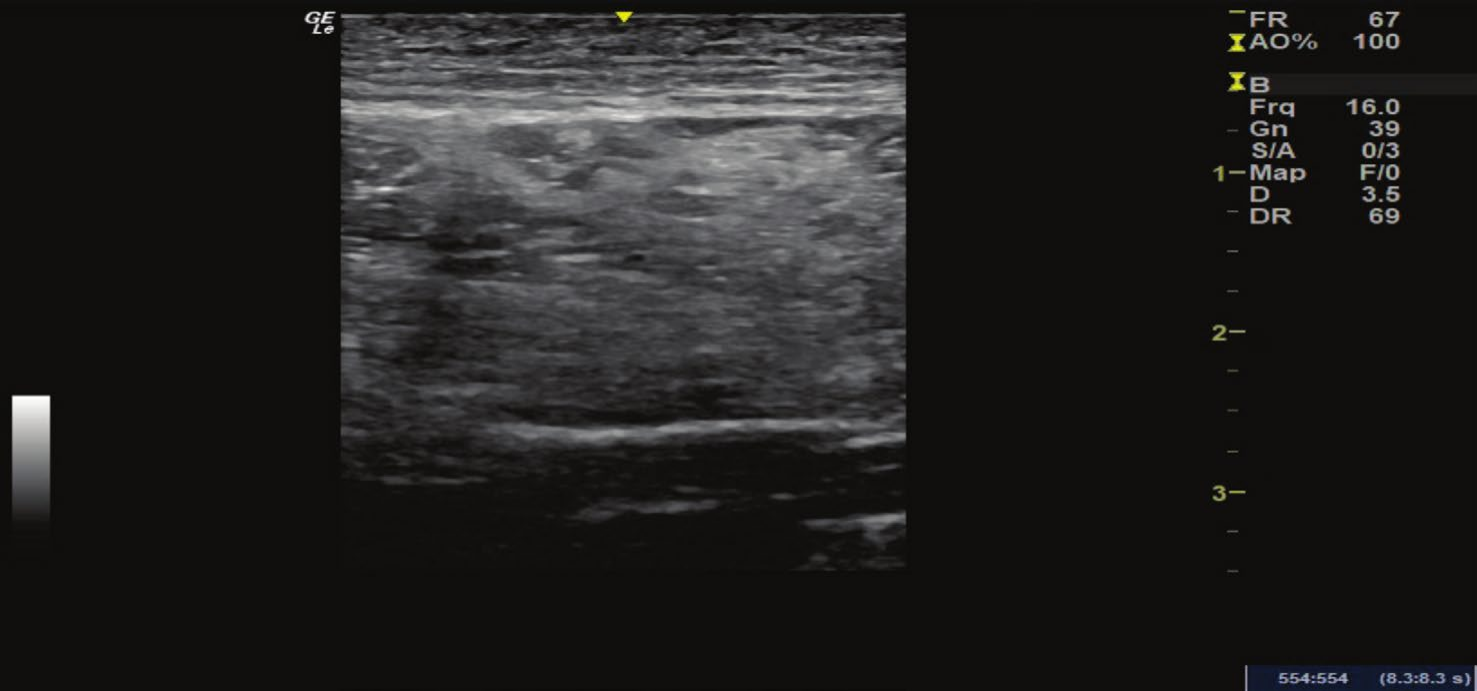
Transverse axis image acquired with a linear transducer (6–15 mHz) demonstrating a global increase in the echogenicity of the gluteal muscles with loss of the usual fibrillar pattern in the transverse axis due to the presence of intramuscular polymethylmethacrylate.

These ultrasonographic signatures—spiculated hyperechoic zones, comet‐tail artifacts, and acoustic shadows—mirror those reported for subcutaneous PMMA but are here demonstrated within the muscle compartment [3]. The obliteration of the muscle's fibrillar architecture likely reflects both the bulk presence of microspheres and associated early collagen deposition, paralleling histologic findings of granulomatous encapsulation and fibroplasia [4, 5]. Accurate recognition of these patterns enables differentiation of PMMA from other fillers and from pathological processes such as panniculitis or fluid collections, thereby facilitating timely management of complications.

Our observations underscore the indispensability of high‐resolution ultrasound for pre‐procedure product diagnostics in patients who already have other fillers and in monitoring possible complications. Given the rising global demand for cosmetic gluteal enhancement [6], practitioners should be familiar with the sonographic hallmarks of intramuscular PMMA to optimize patient safety and to guide intervention when adverse events arise.

In conclusion, subcutaneous PMMA in gluteal augmentation produces distinct ultrasound patterns characterized by poorly defined hyperechoic foci with comet‐tail reverberations and posterior acoustic shadowing. Although global echogenicity augmentation and loss of the normal muscular fibrillar pattern are seen in the intramuscular PMMA, recognition of these features on both linear and convex transducers is critical for accurate assessment and monitoring of patients undergoing permanent filler injections.

## Author Contributions

**Eliza Porciuncula Justo Ducati:** contributed to the search for scientific articles, the writing of this article, the review of the content and the suggestion of revisions, the conduct and analysis of imaging examinations, and the description of ultrasonographic findings. **Ximena Wortsman:** contributed her expertise by reviewing the content and suggesting revisions. **Gabriel de Sousa Lima:** contributed to the search for scientific articles and the writing of this article. **Camila Souza de Araujo Guarinello:** contributed to the search for scientific articles and the writing of this article.

## Conflicts of Interest

The authors declare no conflicts of interest.

## Data Availability

Research data are not shared.
